# Pathogenic characteristics of hand, foot and mouth disease in Shaanxi Province, China, 2010–2016

**DOI:** 10.1038/s41598-020-57807-z

**Published:** 2020-01-22

**Authors:** Yi Xu, Yuan Zheng, Wei Shi, Luyuan Guan, Pengbo Yu, Jing Xu, Lei Zhang, Ping Ma, Jiru Xu

**Affiliations:** 1 Department of Microbiology and Immunology, School of Medicine, Xi’an Jiaotong University, Xi’an, China; 2 Department of Viral Disease Control and Prevention, Shaanxi Center for Disease Control and Prevention, Xi’an, China

**Keywords:** Viral infection, Molecular medicine

## Abstract

Hand, foot, and mouth disease (HFMD) is a common childhood illness caused by enteroviruses. We analyzed the pathogenic characteristics of HFMD in Shaanxi province, China, during 2010–2016. Clinical samples were collected from HFMD cases. Real-time PCR and RT-PCR were used to identify the enterovirus(EVs) serotypes. Viral RNA sequences were amplified using RT-PCR and compared by phylogenetic analysis. Descriptive epidemiological methods were used to analyze. A total of 16,832 HFMD positive cases were confirmed in the laboratory. EV-A71 and CV-A16 were the main pathogens in 2010. EV-A71 was the dominant pathogen in the periods of 2011 to 2012 and 2014, 2016. In 2013 and 2015, other EVs increased greatly, in which CV-A6 was the predominant pathogen. EV-A71 was more frequently detected in deaths and severe cases. Phylogenetic analysis revealed that EV-A71 belonged to the C4a evolution branch of C4 sub-genotype and CV-A16 belonged to the B1a or B1b evolution branch of B1 sub-genotype, whereas CV-A6 strains were assigned to D2 or D3 sub-genotype. The pathogen spectrum of HFMD has changed in 7 years, and the major serotypes EV-A71, CV- A16 and CV- A6 alternated or co-circulated. Long-term surveillance and research of EVs should be strengthened for the prevention and control of HFMD.

## Introduction

Hand foot and mouth disease (HFMD) is a common infectious disease in children caused by human enterovirus (EV)^1–3^. The main clinical manifestations of HFMD are fever, rash on the palms, soles, mouth and buttocks. Most HFMD patients have a good prognosis. A few patients may have complications such as aseptic meningitis, encephalitis, acute flaccid paralysis, neurogenic pulmonary edema and myocarditis. Individual severe children may die due to exacerbations^3–5^. Since the outbreak of HFMD in Anhui Province in March 2008^6^, there has been a widespread epidemic in mainland of China^7–9^. It has become one of the main public health problems which seriously endanger the health of infants and young children.

EVs belong to the family of *Picornaviridae*, *enterovirus* genus^10^. A variety of EVs can cause HFMD. According to its gene and antigen characteristics, EVs can be divided into four species: *EV-A, EV-B, EV-C, and EV-D*. Human enterovirus 71 (EV-A71) and Coxsackievirus A16 (CV-A16) in *EV-A* species are generally considered to be the common and major pathogens causing HFMD^11–13^. In recent years, however, more and more studies have shown that other EVs also play an important role in the outbreak or epidemic of HFMD^14-18^. In particular, CV-A6 has replaced EV-A71 and CV-A16 as the main pathogen for the outbreak or epidemic of HFMD in mainland of China since 2012^19–21^. The change of pathogen spectrum put forward a new challenge to the prevention and control of HFMD.

In this study, we analyzed the pathogenic characteristics of HFMD in Shaanxi province during 2010–2016, and clarified the epidemiological characteristics and pathogen spectrum changes of HFMD. It could provide scientific basis for prevention and control of HFMD in Shaanxi Province.

## Results

### Pathogenic surveillance in shaanxi province during 2010–2016

**Figure 1:**
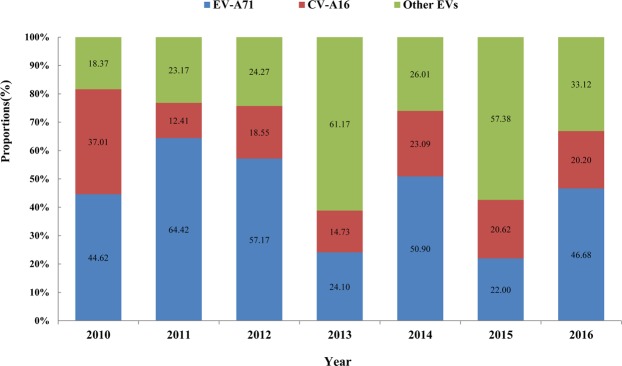
Pathogen proportion of HFMD confirmed cases in Shaanxi Province, China, 2010–2016. The histogram showed the proportion of EVA71, CVA16 and other EVs detected in the confirmed HFMD cases. The proportion of pathogens detected in 7 years has difference.

A total of 392,400 HFMD cases were reported in Shaanxi Province during 2010–2016, including 5469 severe cases and 137 deaths. The average annual incidence rate was 148.5319 per 100,000. The average annual mortality rate and fatality rate were 0.04806 per 100,000 and 2.32%, respectively. A total of 16,832 HFMD positive cases were confirmed in the laboratory, of which 7042 (44.84%) were positive for EV-A71 infection, 3473 (20.63%) were positive for CV-A16 infection and 6317 (37.53%) were other EVs. The proportion of pathogens detected in 7 years was differenced (Fig. 1). In 2010, EV-A71 and CV-A16 were the main pathogens causing HFMD, and the positive rates of EV-A71 and CV-A16 were 44.62% and 37.01%, respectively. In 2011 and 2012, EV-A71 was the dominant pathogen which the positive rates were 64.42% and 57.17%, respectively. In 2013, the pathogen spectrum of HFMD changed greatly, and the proportion of other EVs increased significantly as high as 61.17%. In 2014, the dominant position of EV-A71 was recovered to 50.91%. In 2015, HFMD was still mainly caused by other EVs, accounting for 57.38%, and in 2016, EV-A71 became the dominant pathogen again, accounting for 46.68%.

**Figure 2:**
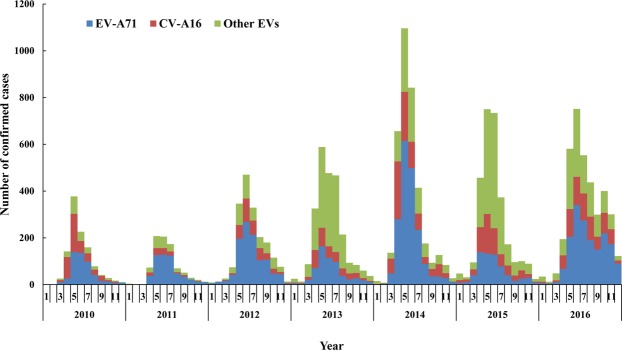
Monthly distribution of pathogen proportion of HFMD confirmed cases in Shaanxi Province, China, 2010–2016. The histogram showed the distribution of EVA71, CVA16 and other EVs detected in each month, the number of confirmed HFMD cases appeared seasonal distribution with the peaks in April to July.

During 2010–2016, HFMD cases were reported monthly in Shaanxi Province, China, the incidence peak appeared from April to July. The peak of positive pathogen was also detected in April to July, accounting for 69.05% of the total number of confirmed samples. Monthly distribution of the pathogens was illustrated in Fig. 2, the epidemic pathogen in different months varied greatly in 7 years. From April to May and September 2010, CV-A16 was the predominant pathogen, and the rest of 2010 and the whole year of 2011 and 2012, the main epidemic pathogen was EV-A71. From January 2013 to January 2014, other EVs became the dominant pathogen. In February and May to August 2014, the epidemic pathogen was still predominant in EV-A71, but in the periods of March to April and September to October, EV-A71 and CV-A16 were the co-epidemic pathogens. From November 2014 to March 2016, other EVs became the dominant pathogen again, and EV-A71 returned to be the main epidemic pathogen in the rest of 2016.

**Figure 3:**
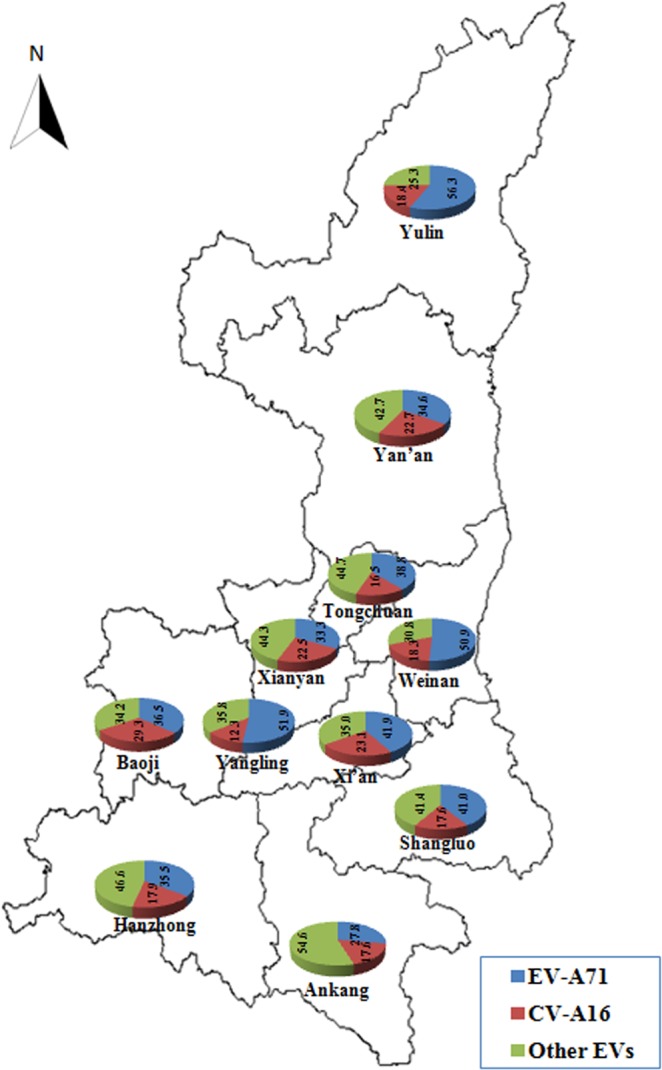
Geographic distribution of pathogen proportion of HFMD confirmed cases in Shaanxi Province, China, 2010–2016. The map shows the proportion of EVA71, CVA16 and other EVs detected in the confirmed HFMD cases in each city. The proportion of pathogens detected in 11 cities has difference.

From 2010 to 2016, most of the cases of HFMD in Shaanxi Province were reported by Xi’an City, Xianyang City and Weinan City. The number of cases in the three cities accounted for 40.95%, 16.88% and 12.02% of the total cases, respectively. The proportion of pathogens detected in 11 cities was differenced (Fig. 3). EV-A71 was the main pathogen causing HFMD in Xi’an City, Weinan City, Yulin City and Yangling District. In Xianyang City, Hanzhong City, Ankang City, Tongchuan City and Yan’an City, the dominant pathogen of HFMD was other EVs, while in Baoji City and Shangluo City, EV-A71 and other EVs were the co-epidemic pathogens.

Among the HFMD positive cases confirmed in laboratory during 2010–2016, there were 10244 males and 6588 females, with an average annual male to female ratio of 1.55:1. The proportion of pathogens in the gender was shown in Table 1, there was no significant difference in the pathogen proportion of different gender (χ^2^ = 0.133, *P* > 0.05). HFMD positive cases mainly concentrated in children aged 1 to 3 years old, accounting for 73.12%, of which the proportion of 1- year-old group was the highest (31.99%). There was significant difference in the proportion of pathogens in different age groups(χ^2^ = 231.765, *P* <0.001).

**Table 1:** Population distribution of pathogen proportion of HFMD confirmed cases in Shaanxi Province, China, 2010–2016.

Variable	No. of positive cases	Enterovirus serotype			χ^2^	*P* value
		EV-A71 (%)	CV-A16 (%)	Other EVs (%)		
Gender					χ^2^ = 0.133	0.936
male	10244	4280 (41.09)	2108 (20.24)	3856 (37.02)		
famale	6588	2762 (41.92)	1365 (20.72)	2461 (37.36)		
age group (years)					χ^2^ = 231.765	0.000
0~	1754	608 (34.66)	316 (18.02)	830 (47.32)^*^		
1~	5384	2147 (39.88)	964 (17.90)	2273 (42.22)^**#**^		
2~	3797	1598 (42.09)	834 (21.96)	1365 (35.95)		
3~	3127	1410 (45.09)	727 (23.25)	990 (31.66)		
4~	1486	693 (46.64)	331 (22.27)	462 (31.09)		
≥5	1284	586 (45.64)	301 (23.44)	397 (30.92)		

^*^Among children under 1 year old, the most frequently detected in other EVs was CV-A6 (46.56%), followed by CV-A10 (20.61%), the remaining 11 serotypes were less than 2%.

^**#**^Among children in the 1–2 age group, the most frequently detected was CV-A6 (42.90%), followed by CV-A10 (26.73%), the remaining 12 serotypes were less than 2%.

**Figure 4:**
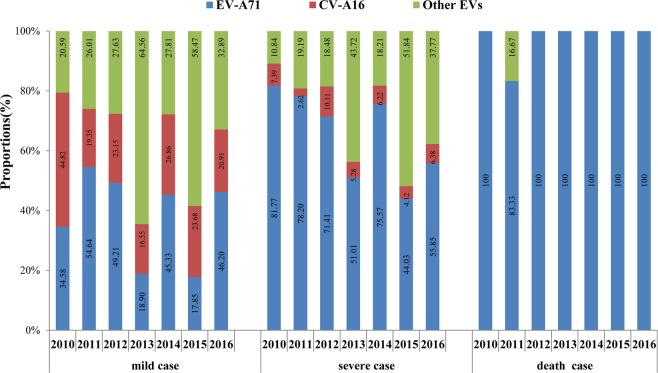
Pathogen proportion in different case types of HFMD in Shaanxi Province, China, 2010–2016. The histogram showed the proportion of EVA71, CVA16 and other EVs detected in different case types of HFMD. EV-A71 was more frequently detected in death and severe cases than in mild cases.

Of all laboratory-confirmed cases during 2010–2016, there were 13,882 mild cases, 2886 severe cases and 64 deaths. The positive rates of EV-A71, CV-A16 and other EVs in mild cases were 36.62%, 23.71% and 39.67%, respectively. In severe cases, the positive rate of EV-A71 was 65.70%, while CV-A16 and other EVs were 6.27% and 28.03% respectively. Furthermore, the positive rate of EV-A71 reached 98.43% in all the deaths except 1 cases of other EVs infection (Fig. 4). There was significant difference in the proportion of pathogens in different case types. EV-A71 was more frequently detected in deaths and severe cases than in mild cases, while CV-A16 was more frequently detected in mild cases than in severe cases, no CV-A16 was detected in deaths.

### Serotypes of other EVs

In this study, a total of 709 other EVs were identified during 2010–2016, including 19 serotypes, belonging to species A and B of EVs. Among the other EVs, the most frequently detected was CV-A6, accounting for 41.47%, followed by CV-A10 (26.66%), CV-A2 (2.54%), CV-A4 (1.55%), Echo6 (1.16%) and CV-B1 (1.13%), respectively. The remaining 13 serotypes were CV-A5, CV-A12, CV-A14, CV-B3, CV-B4, CV-B5, Echo3, Echo7, Echo8, Echo9, Echo11, Echo16, Echo18, respectively, less than 1%. In 2013 and 2015, CV-A6 was the main serotype accounting for 40.85% and 39.49%, respectively, which exceeded the level of EV-A71 in the same year and became the dominant pathogen of HFMD in that year.

### Phylogenetic analyses

**Figure 5:**
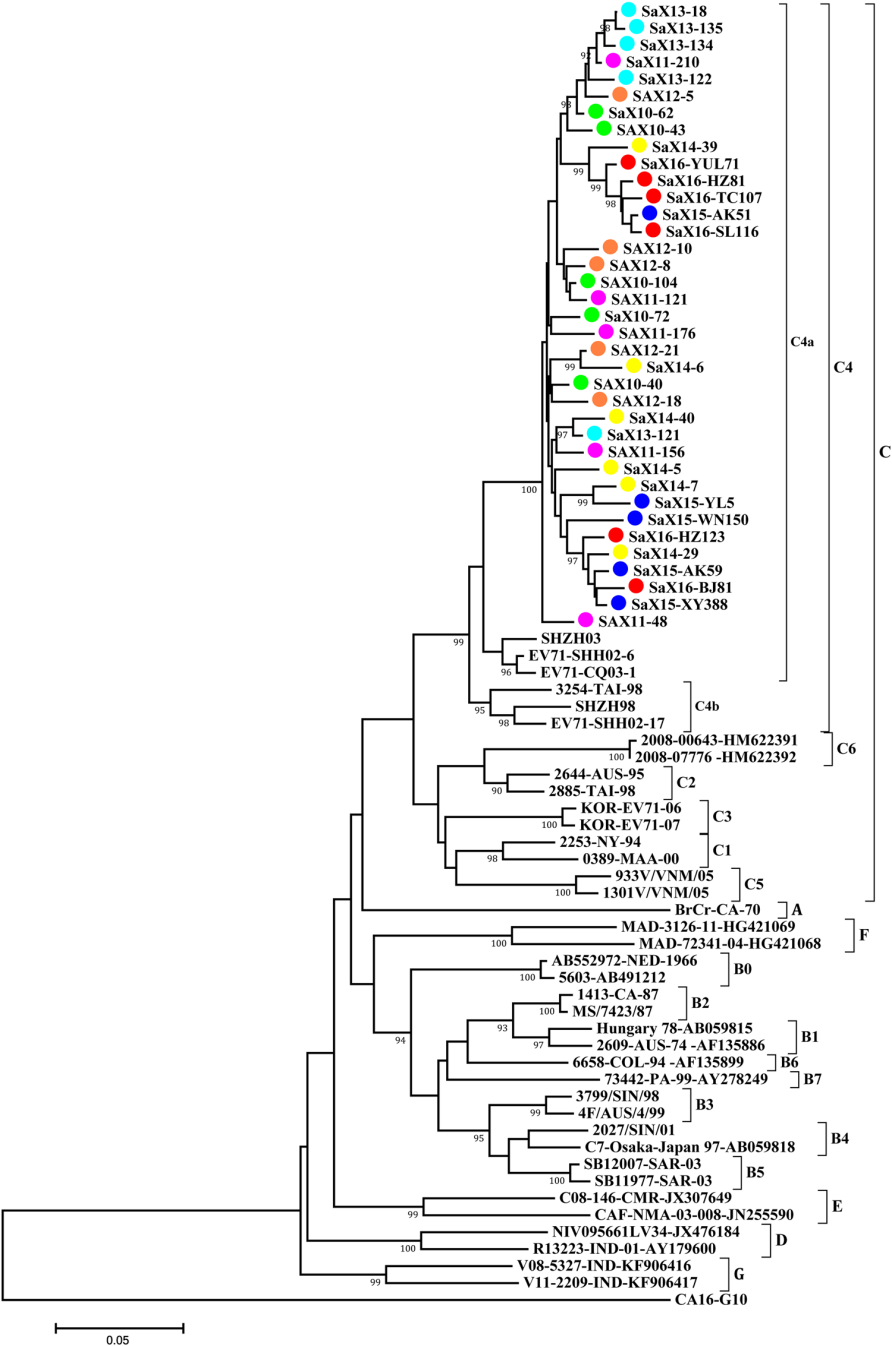
Phylogenetic tree of VP1 region of EV-A71 Strains Isolated from HFMD in Shaanxi Province, China, 2010–2016. The neighbor-joining method was used to construct the tree. The phylogenetic tree was determined for 1000 replicates. Only strong bootstrap values (≥90%) were shown. The viruses isolated from Shaanxi province were labeled with different colour solid circle, in which 2010 (green), 2011 (purple), 2012 (orange), 2013 (wathet), 2014 (yellow), 2015 (blue) and 2016 (red). The 40 reference strains were obtained from the US National Center for Biotechnology Information’s Genbank. BrCr(U22521) was the prototype strain of EV-A71.

The entire VP1 genes of EV-A71, CV-A16 and CV-A6 which the major serotypes of HFMD were sequenced and analyzed. According to the type of cases and different years, 37 strains of EV-A71, 36 strains of CV-A16 and 29 strains of CV-A6 were selected. The results showed that the nucleotide homology of 37 EV-A71 isolates from Shaanxi province was 94.2–99.7%, and the amino acid homology was 98.0–100%. The nucleotide and amino acid homology with EV-A71 prototype strain (BrCr) were 80.6–82.7% and 92.3–94.9%, respectively. The nucleotide and amino acid homology with the representative strain of C4 sub-genotype (SHZH98) were 90.8–92.6% and 96.3–97.6%, respectively. The phylogenetic tree was constructed by selecting the genotypes and sub-genotypes of EV-A71 from GenBank. The isolated strains of Shaanxi EV-A71 gathered into clusters. All of them were located on the C4a evolutionary branch of C4 sub-genotype and belonged to the same transmission chain (Fig. 5).

**Figure 6:**
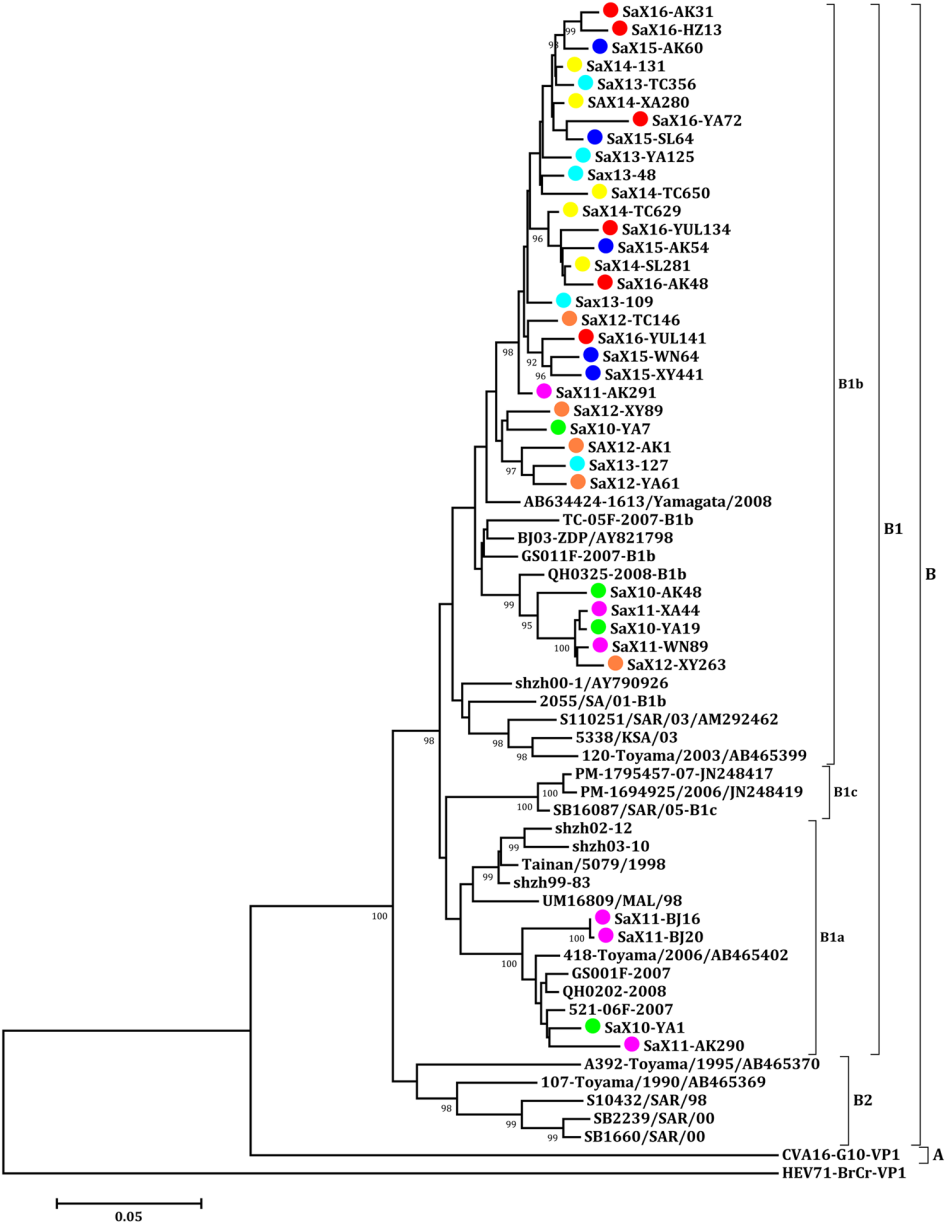
Phylogenetic tree of VP1 region of CV-A16 Strains Isolated from HFMD in Shaanxi Province, China, 2010–2016. The neighbor-joining method was used to construct the tree. The phylogenetic tree was determined for 1000 replicates. Only strong bootstrap values (≥90%) were shown. The viruses isolated from Shaanxi province were labeled with different colour solid circle, in which 2010 (green), 2011 (purple), 2012 (orange), 2013 (wathet), 2014 (yellow), 2015 (blue) and 2016 (red). The 27 reference strains were obtained from the US National Center for Biotechnology Information’s Genbank. G-10(U05876) was the prototype strain of CV-A16.

The nucleotide and amino acid homologies of 36 isolates of CV-A16 from Shaanxi province were 89.2–99.9% and 98.3–100%, respectively. The nucleotide and amino acid homologies with CV-A16 prototype strain (G-10) were 80.6–82.7% and 92.3–94.9%, respectively. The phylogenetic tree was constructed between Shaanxi CV-A16 isolates and reference genotypic strains from GenBank. It can be seen that Shaanxi isolates clustered with the B1 sub-genotype of B genotype, and B1 sub-genotype was divided into two major evolutionary branches B1a and B1b. There were some differences in nucleotide level between the two evolutionary branches. The B1a evolutionary branch contained isolates from Shaanxi province during 2010–2011. Most of CV-A16 isolates belonged to B1b evolutionary branch since 2012 (Fig. 6).

**Figure 7:**
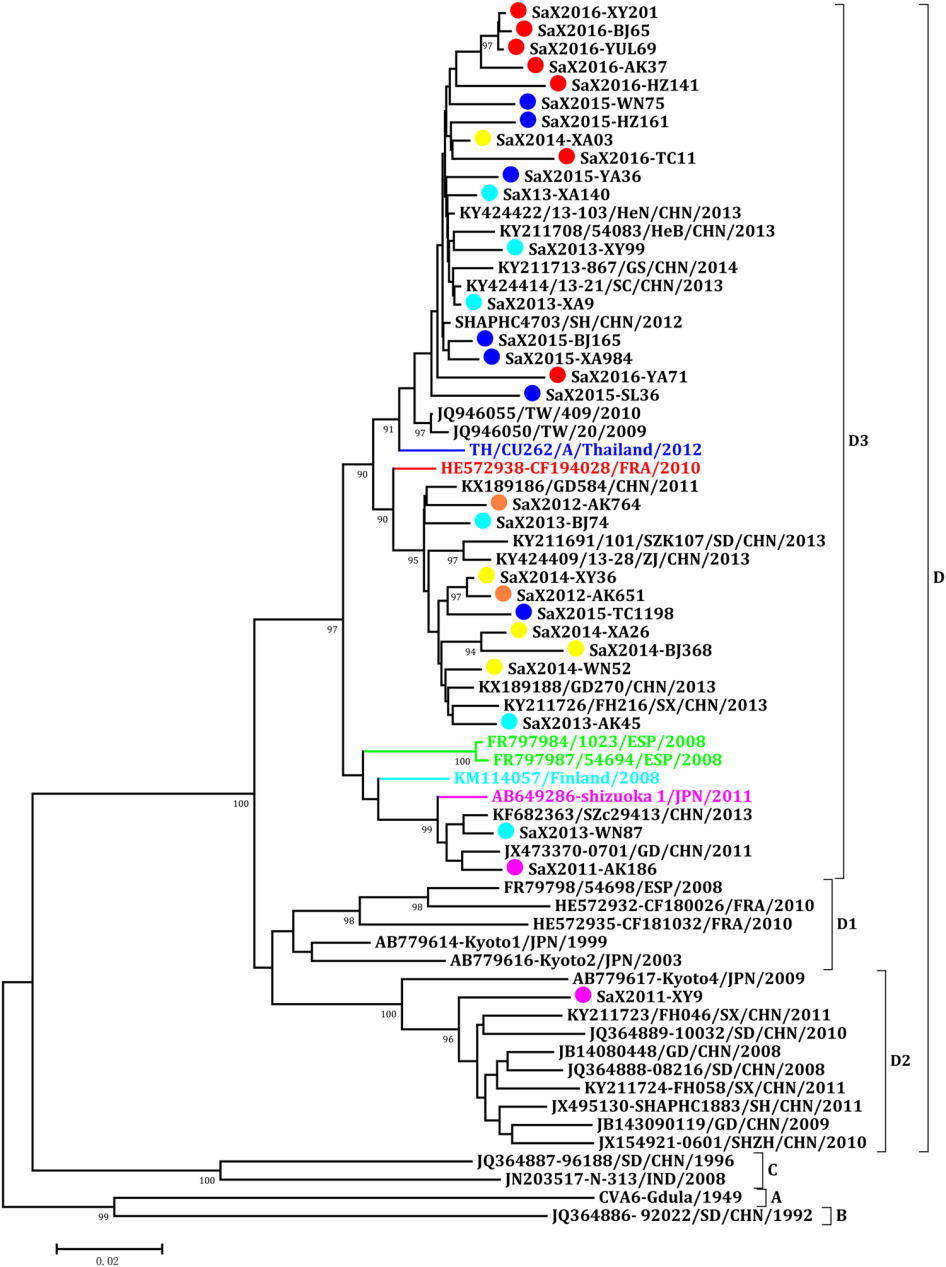
Phylogenetic tree of VP1 region of CV-A6 Strains Isolated from HFMD in Shaanxi Province, China, 2010–2016. The neighbor-joining method was used to construct the tree. The phylogenetic tree was determined for 1000 replicates. Only strong bootstrap values (≥90%) were shown. The viruses isolated from Shaanxi province were labeled with different colour solid circle, in which 2010(green), 2011(purple), 2012 (orange), 2013(wathet), 2014(yellow), 2015(blue) and 2016 (red). The 38 reference strains were obtained from the US National Center for Biotechnology Information’s Genbank. Gdula(AY421764) was the prototype strain of CV-A6. The branches of sequences from other international strains were marked with different colors, including France (red), Spain (green), Finland (wathet), Japan (purple) and Thailand (blue).

Homology comparison and phylogenetic analysis of CV-A6 isolates in Shaanxi Province and reference CV-A6 strains from GenBank were carried out. The nucleotide and amino acid sequence homologies of Shaanxi CV-A6 isolates were 93.6–99.9% and 95.4–100%, respectively. The nucleotide and amino acid homologies with CV-A6 prototype strain (Gdula) were 81.6–83.7% and 93.1–95.7%, respectively. The phylogenetic tree showed that CV-A6 could be divided into 4 genotypes A to D, and genotype D could be further divided into 3 sub-genotypes D1-D3. Most of CV-A6 isolates in mainland of China belonged to the D2 and D3 sub-genotypes of D genotype. Shaanxi CV-A6 isolates clustered with the D3 sub-genotype, except for 1 strain (XY09/SaX/CHN/2011) in 2011, which located on the D2 sub-genotype. Shaanxi CV-A6 strains formed at least 3 small transmission chains, which were closely related to strains isolated from Henan, Sichuan, Gansu, Shandong and Guangdong provinces of China in recent years (Fig. 7).

## Discussion

HFMD is a worldwide infectious disease, most countries and regions have epidemic coverage^23–28^. As a common infectious disease in children, it has strong infectivity, complex transmission route, strong epidemic intensity and spread rapidly. A total of 392,400 HFMD cases from NNDRS were reported in Shaanxi Province during 2010–2016, and the average annual incidence rate was 148.5319/100,000, the incidence of HFMD showed a trend of increasing year by year.

The incidence of HFMD in Shaanxi Province was obviously seasonal, and the peak incidence concentrated in April to July, consistent with other reports in China^29,30^. Some studies suggested that the seasonal epidemic of HFMD is related to climatic factors such as precipitation, sunshine duration, temperature and pressure. The hot and humid environment in spring and summer is good for the survival of EVs^3,31^. Moreover, People outdoor activities make the chance of infection increased, which lead to the frequent occurrence of HFMD cases. HFMD positive cases were mainly concentrated in children under 3 years old. It is possible that the immune system of children in this age group is not perfect, their resistance to infection is weak and health habits have not yet been developed, so children are generally susceptible to EVs.

The etiological surveillance of HFMD in Shaanxi Province revealed that the type of pathogens were differenced during 2010–2016. In 2010, the pathogen of HFMD was the co-epidemic of EV-A71 and CV-A16. In the period of 2011 to 2012, EV-A71 was the major dominant pathogen. In 2013, the proportion of CV-A6 was significantly increased to 40.85%, exceeding that of EV-A71 becoming the dominant pathogen. In 2014, EV-A71 returned to the dominant pathogen of 50.91%. In 2015, CV-A6 was still the main pathogen of HFMD, accounting for 39.49%. In 2016, EV-A71 became the dominant pathogen of HFMD again, accounting for 46.68%. The pathogen spectrum of HFMD in Shaanxi Province varied, the main serotypes appeared alternately, or simultaneously epidemic, presenting a dynamic change.

According to the analysis of the pathogenic proportion of different types of HFMD cases, it can be seen that the positive rates of EV-A71 in severe and dead cases were 65.70% and 98.43%, respectively. EV-A71 was the major pathogen causing severe and dead cases of HFMD. It has been reported that the positive rate of EV-A71 is closely related to the occurrence of severe and dead cases^32,33^. When EV-A71 was the dominant pathogen in a certain year, the severe and dead cases were higher than those in the other year. EV-A71 is a neurotropic virus that causes aseptic meningitis, encephalitis, neurogenic pulmonary edema, other central nervous system diseases, and even causes death^34^. There were 64 laboratory-confirmed deaths in this study, 63 of the 64 deaths died of neurological complications (such as brain stem encephalitis, aseptic meningitis, encephalomyelitis) or cardiopulmonary complications (neurogenic pulmonary edema, pulmonary hemorrhage or cardiopulmonary failure) caused by EV-A71 infection. Therefore, we should pay more attention to the epidemic of EV-A71 in HFMD. When the positive rate of EV-A71 appears a high level, it is necessary to predict and warn the occurrence of severe and dead cases in time.

The VP1 coding region contains many important neutralization epitopes and was demonstrated to help identify different serotypes of EVs^35^. A phylogenetic dendrogram based on the entire VP1 sequences of EV has been used for discrimination of genotypes. This study conducted the entire VP1 sequence-based phylogenetic analyses of 3 predominant serotypes of EV-A71, CV-A16 and CV-A6 isolated from Shaanxi province during 2010–2016. The results showed that the nucleotide and amino acid homologies of Shaanxi EV-A71 strains were 94.2–99.7% and 98.0–100%, respectively, which were highly homologous to the representative strain of C4 sub-genotype in China. According to the nucleotide sequence difference of VP1, EV-A71 can be divided into 7 genotypes (A-G), in which B genotype includes 8 sub-genotypes (B0 to B7), and C genotype includes 6 sub-genotypes (C1 to C6). Since the first strain of EV-A71 virus (genotypes A) was isolated in the United States in 1969^1^, there has been an epidemic of HFMD caused by EV-A71 infection in the world. B1, B2 and C1, C2 sub-genotypes spread in European and American countries and Australia, Japan from 1970s to 1990s^24–26^. After 1997, B3~B5 and C3~C6 sub-genotypes began to appear and spread in Southeast Asia^13,14,23^. EV-A71 virus isolated in India in 2001 was classified as genotypes D and G, while genotypes E and F were the main epidemic in Africa^27^. In the process of continuous evolution of EV-A71 virus, each genotype has different time and regional distribution characteristics. At present, the main epidemic sub-genotypes of EV-A71 in Asia were C2, C4, C5 and B5. B5 and C2 are prevalent in Malaysia and Singapore^23^, C2, C4 and B5 are mainly popular in Japan^26^, and C5 is prevalent in Vietnam^28^. C4 sub-genotype was first isolated in mainland of China in 1998, and this sub-genotype has been prevalent until now. C4 sub-genotype can be divided into two branches: C4a and C4b. The main epidemic strains from 2008 to 2004 belonged to C4b branch, and the C4a branch has the dominant genotype since 2004^36^. All of Shaanxi EV-A71 strains formed a gene cluster in the phylogenetic tree, which were located on the C4a branch of C4. Shaanxi EV-A71 strains have the same evolutionary origin as that strains epidemic in mainland of China in recent years. EV-A71 virus has been widely spread in Shaanxi Province.

Based on the phylogenetic analysis of nucleotide sequences in the VP1 region, CV-A16 was divided into two genotypes A and B. A genotype was only the prototype strain and was isolated in South Africa in 1951. B genotype was divided into two sub-genotypes (B1~B2), and B1 sub-genotype was further divided into three branches B1a, B1b and B1c^37^. The change of epidemic genotype of CV-A16 is closely related to epidemic time, but there is no difference in epidemic region. A genotype was no longer popular after the 1990s. B2 sub-genotype has not been reported since 2000^26^. At present, B1 sub-genotype was the dominant genotypes circulating in the Asia-Pacific region, with the alternation of B1a and B1b branches. B1c only occurred in Malaysia during 2005–2007 and HFMD outbreak in France in 2010^38^. The epidemic CV-A16 in mainland of China belongs to the B1a and B1b branches of B1, which is closely related to the strains in Japan, Vietnam, Malaysia and Taiwan, indicating that CV-A16 in China co-evolved with the strains in the surrounding areas. All of Shaanxi CV-A16 isolates clustered with B1 sub-genotype, and two evolutionary branches of B1a and B1b were alternately epidemic. The strains between 2010 and 2011 were located on the B1a branch, while all of strains isolated after 2012 were belonged to the B1b branch. It indicated that the B1b branch of CV-A16 strains was dominant and had gradually replaced B1a as endemic epidemic virus in Shaanxi Province.

CV-A6 associated HFMD outbreaks have taken place in the worldwide since the first reported in Finland in 2008^16^, it was circulated in France^17^, Spain^18^, and other European countries from 2009 to 2011. In Asia, HFMD outbreaks caused by CV-A6 occurred during the same period, including Singapore in 2009^14^, Japan in 2011^39^, Thailand in 2012^40^, and mainland of China in 2013^19–21^. The phylogenetic tree showed that CV-A6 could be divided into 4 genotypes A to D, and D genotype could be further divided into 3 sub-genotypes D1-D3. D1 was composed of strains isolated in Japan in 1999 and in France and Spain from 2008 to 2010, D2 was composed of strains isolated in Japan and mainland of China from 2008 to 2011, while D3 was composed of strains isolated in Finland, Spain, France, Japan, and China from 2011 to 2016. Most of the CV-A6 isolates epidemic in China after 2012 belonged to the D3 sub-genotype, and clustered with the international isolates, the results revealed that D3 was the predominant sub-genotype circulated and spread in Europe and Asia during recent years. D3 sub-genotype may have strong transmission ability and virulence in the process of continuous circulating^41^, which lead to the outbreaks of HFMD in Europe, Southeast Asia and mainland of China. Shaanxi CV-A6 isolates clustered with the D3 sub-genotype, and formed at least 3 small transmission chains, which co-evolved with the strains from Henan, Gansu, Sichuan, Shandong and Guangdong provinces of China.

Enterovirus (EVs) has many serotypes, and there is no cross-immunization between different EVs serotypes, so when the immune barrier is established by one serotype of EVs, the epidemic of this EVs will be weakened. EV-A71 and CV-A16 were the main epidemic serotypes of HFMD in Shaanxi provinces from 2010 to 2012 and 2014, the susceptible population obtained protective antibodies through contact or cross-infection in the epidemic period, which blocked the transmission chain of the virus and declined the transmission power of EV-A71 and CV-A16. Very coincidentally, the prevalence of CV-A6 associated HFMD in on the rise in mainland of China in the same period. In recent years, CV-A6 outbreaks have been reported in many countries around the world. Since the outbreak of HFMD caused by CV-A6 in Finland in 2008, HFMD with CV-A6 as the main pathogen has also occurred in many other countries in the world. From 2009 to 2011, CV-A6 was widely spread in France, Spain and other European countries. And since 2009, CV-A6 has become one of the most important pathogens of HFMD in Asian countries, including Singapore, Japan, Thailand and mainland of China. In phylogenetic tree, the epidemic CV-A6 strains in China after 2012 were clustered together with the isolates of Japan, Thailand, Finland, Spain and France countries, and the strains were closely related to each other. This finding suggested that the emerging strains of CV-A6 might have a common origin and circulated and transmitted in Europe, Southeast Asia and mainland of China. Therefore, we have reason to believe that CV-A6 has been popular and became the dominant pathogen causing HFMD in Shaanxi provinces in 2013 and 2015 due to the importation of European CV-A6.

This study found that the pathogen spectrum of HFMD in Shaanxi Province has changed, and the major serotypes EV-A71, CV-A16 and CV-A6 alternated or co-epidemic. Therefore, it is necessary to strengthen the long-term surveillance and research of EVs, to understand the epidemiological characteristics and genetic variation of the EVs so as to provide a scientific basis for the early warning and prevention and control of HFMD.

## Methods

### Sample collection

HFMD surveillance data reported from the National Notifiable Disease Reporting System (NNDRS) were collected. All the patients were diagnosed by the Ministry of Health diagnostic criteria. Generally, children who had fever or one of the following features: maculopapular or vesicular rash on the palms and/or soles, or vesicles and ulcers around the uvula, were diagnosed as HFMD-positive. Severe manifestations of the disease included encephalitis, meningitis, acute flaccid paralysis or even death. According to the guidelines for prevention and control of HFMD issued by the Ministry of health in China in 2009, the selection criteria for cases in this study are as follows: at least 5 mild cases were collected each month in counties (districts), and all severe and dead cases were collected. Samples of HFMD cases were collected from the sentinel surveillance hospitals in Shaanxi Province during 2010–2016. Clinical samples were processed in accordance with the HFMD Laboratory Manual (4th edition, 2010).

### Nucleotide acid detection

Viral RNA was extracted using QIAamp Viral Mini RNA Extraction kit (Qiagen, CA, USA). EV-A71, CV-A16 and other EVs types were detected by real-time fluorescence quantitative reverse transcription polymerase chain reaction (RT-PCR). The RT-PCR kit was produced by Shuoshi Biotechnology Company (Jiangsu, China), the experimental operation was carry out according to the manufacturer’s instructions. The instrument was ABI 7500 real-time fluorescence quantitative PCR instrument.

### Viral isolation

The positive samples were inoculated into human rhabdomyosarcoma (RD) and human laryngeal epidermoid carcinoma (HEp-2) cell lines for virus propagation and purification. If typical cytopathic effect (CPE) of EVs was observed, the virus culture supernatant was collected and frozen at 20 °C.

### VP1 amplification and sequencing

Viral RNA was extracted from the positive culture supernatants. Reverse transcription polymerase chain reaction (RT-PCR) was performed to amplify the entire VP1 gene using PrimeScript One Step RT-PCR Kit Ver. 2 (TaKaRa, Dalian, China). The primers have been reported previously^22^. The reaction conditions were: 50 °C 30 min, 94 °C 2 min; 94 °C 30 S, 55 °C 30 S, 72 °C 1 min, 35 cycles; extension 10 min at 72 °C. The PCR products were detected with 1.5% agarose gel electrophoresis, and the positive products were purified using the QIAquick PCR Purification Kit (Qiagen, Germany). The amplicons were bi-directionally sequenced using ABI 3500 Genetic Analyser (Applied Biosystems, USA).

### Phylogenetic analysis

The nucleotide sequences were assembled using Squencher software (version 4.0). DNAstar software was used for homology sequence analysis. Sequence alignment was conducted using MEGA software (version 5.0) and phylogenetic tree was constructed for the VP1 sequences by the neighbour-joining method based on the Kimura 2-parametermodel, with 1000 bootstrap replicates. All the reference strains used were retrieved from the GenBank database of the National Center for Biotechnology Information (NCBI).

### Statistical analysis

Statistical analysis was performed with SPSS software (version 18.0). Descriptive statistics was used to describe epidemiological characterizations. *X*^2^ test was used for categorical variable. P < 0.05 was considered statistically significant.

### Ethics statement

This study did not involve human participants or human experimentations, the only human materials used were the throat swab and stool samples collected from suspected HFMD patients for public health purposes. All information collected from the patients was approved and written informed consents were obtained from the patients. The study was approved by the Ethics Review Committee of the Shaanxi province CDC and was carried out in accordance with the approved guidelines.
